# Naturalist's Monthly Report

**Published:** 1812-04

**Authors:** 


### NATURALIST'S MONTHLY REPORT.

### FEBRUARY.

THAWING MONTH.

The rivers swell
Of bonds impatient, sudden from the hills,
O'er rocks and woods, in broad brown cataracts,
A thousand snow-fed torrents shoot at once.

DURING the present month there has scarcely been any cessation of rainy and stormy weather. Of the twenty-nine days we had only eight in which there was no rain, viz. the 4th, 8th, 9th, 10th, 11th, 15th, 18th, and 20th. There were fresh gales on the 1st, 6th, 8th, 13th, 19th, 23d, 26th, and 29th; strong gales on the 2d, 7th, 12th, 17th, 21st, and 22d; and hard gales on the 14th and 25th.

From the 1st to the 3d the wind was south-east; on the 4th south-west; on the 5th and 6th easterly; on the 7th and 8th westerly; on the 9th variable; from the 10th to the 12th south-west; on the 13th north-west; from the 14th to the 18th westerly; on the 19th south-west; on the 20th variable; from the 21st to the 23d south-west; on the 24th north; from the 25th to the 28th westerly; and on the 29th south-west.

February 4th. Some of the early spring flowers are beginning to appear; viz. in the gardens, the crocuses, snow-drops, and violets; and in the fields the ivy-leaved veronica *(veronica hederæfolia)*, coltsfoot *(tussilago farfara)*, and purple dead-nettle *(lamium purpureum)*.

The

The hedge-sparrow, lark, and chaffinch, sing.

February 7th. The flowers of the mezereon begin to expand. The leaves of the ivy fall.

Earth-worms come out of their holes.

Rooks begin to employ themselves in repairing and building their nests.

February 9th. The filbert-trees are in full bloom. The barren strawberry *(fragaria sterilis)*, procumbent speedwell *(veronica agrestis)*, yew-tree *(taxus baccater)*, and green helebore *(helleborus viridis)*, are also in flower.

February 11th. The sulphur-colored butterfly *(papilio rhamnus)* appears. The thrush, blackbird, and lark, are heard; and several species of insects, induced by the warmth of the day, are seen flying about, and sporting in the air.

February 15th. Primroses are in flower.

The hedge snails, viz. *helix nemoralis* and *helix hortensis*, crawl forth from their places of concealment. In gardens also the different kinds of slugs crawl out in the night, and devour the leaves of lettuces, and other esculent plants.

February 20th. This was a most delightful day. The birds sang as loudly and melodiously as in the middle of spring. Three or four kinds of butterflies were seen flitting about the hedges and along the roads. I observed also several species of beetles, and amongst the rest the common lady-bug *(coccinella septem punctata)*.

Butcher's broom *(ruscus aculeatus)* is in flower.

February 21st. The rivers and brooks have overflowed their banks.

There was much lightning in the night.

February 22d. A cock-chaffer was dug out of the ground, in a perfect state.

In the night there was a tremendous storm of wind and rain, accompanied with thunder and lightning.

February 26th. In sheltered gardens the bloom of some of the fruit-trees begins to open. The rhubarb appears above the ground.

The night was frosty.

February 28th. This morning the weather cleared up and was fine. The bees were busily employed about the opening catkins of a few of the willow trees, which, from their sheltered situations, were more forward in their flowering than others.

A leveret nearly as large as a half grown rabbit, and apparently a fortnight or three weeks old, was this day caught.

Daffodils *(narcissus pseudo narcissus)* are in flower.

In consequence of the great quantity of rain that has fallen in the course of the month, the rivers have been much higher, and the floods of much longer continuance than usual. Great numbers of lambs in this neighbourhood have died, owing to the wetness of the season.

*Hampshire.*

METEO-

